# Deficient or Excess Folic Acid Supply During Pregnancy Alter Cortical Neurodevelopment in Mouse Offspring

**DOI:** 10.1093/cercor/bhaa248

**Published:** 2020-09-30

**Authors:** Angelo Harlan De Crescenzo, Alexios A Panoutsopoulos, Lyvin Tat, Zachary Schaaf, Shailaja Racherla, Lyle Henderson, Kit-Yi Leung, Nicholas D E Greene, Ralph Green, Konstantinos S Zarbalis

**Affiliations:** Department of Pathology and Laboratory Medicine, University of California, Davis, CA 95817, USA; Institute for Pediatric Regenerative Medicine, Shriners Hospitals for Children, Northern California, 2425 Stockton Boulevard, Sacramento, CA 95817, USA; Department of Pathology and Laboratory Medicine, University of California, Davis, CA 95817, USA; Institute for Pediatric Regenerative Medicine, Shriners Hospitals for Children, Northern California, 2425 Stockton Boulevard, Sacramento, CA 95817, USA; Department of Pathology and Laboratory Medicine, University of California, Davis, CA 95817, USA; Department of Pathology and Laboratory Medicine, University of California, Davis, CA 95817, USA; Institute for Pediatric Regenerative Medicine, Shriners Hospitals for Children, Northern California, 2425 Stockton Boulevard, Sacramento, CA 95817, USA; Department of Pathology and Laboratory Medicine, University of California, Davis, CA 95817, USA; Institute for Pediatric Regenerative Medicine, Shriners Hospitals for Children, Northern California, 2425 Stockton Boulevard, Sacramento, CA 95817, USA; UCL Great Ormond Street Institute of Child Health, University College London, London, UK; UCL Great Ormond Street Institute of Child Health, University College London, London, UK; Department of Pathology and Laboratory Medicine, University of California, Davis, CA 95817, USA; Department of Pathology and Laboratory Medicine, University of California, Davis, CA 95817, USA; Institute for Pediatric Regenerative Medicine, Shriners Hospitals for Children, Northern California, 2425 Stockton Boulevard, Sacramento, CA 95817, USA; MIND Institute, University of California, Davis, CA 95817, USA

**Keywords:** cortical development, folate metabolism, mouse, neurogenesis, projection neurons

## Abstract

Folate is an essential micronutrient required for both cellular proliferation through de novo nucleotide synthesis and epigenetic regulation of gene expression through methylation. This dual requirement places a particular demand on folate availability during pregnancy when both rapid cell generation and programmed differentiation of maternal, extraembryonic, and embryonic/fetal tissues are required. Accordingly, prenatal neurodevelopment is particularly susceptible to folate deficiency, which can predispose to neural tube defects, or when effective transport into the brain is impaired, cerebral folate deficiency. Consequently, adequate folate consumption, in the form of folic acid (FA) fortification and supplement use, is widely recommended and has led to a substantial increase in the amount of FA intake during pregnancy in some populations. Here, we show that either maternal folate deficiency or FA excess in mice results in disruptions in folate metabolism of the offspring, suggesting diversion of the folate cycle from methylation to DNA synthesis. Paradoxically, either intervention causes comparable neurodevelopmental changes by delaying prenatal cerebral cortical neurogenesis in favor of late-born neurons. These cytoarchitectural and biochemical alterations are accompanied by behavioral abnormalities in FA test groups compared with controls. Our findings point to overlooked potential neurodevelopmental risks associated with excessively high levels of prenatal FA intake.

## Introduction

Proper organization of the cerebral cortex depends on the undisturbed birth and migration of cortical neurons during prenatal neurogenesis. Any disturbance of this process may result in cortical malformations that can lead to a range of neurodevelopmental disorders. For instance, neuronal migration defects may lead to intractable childhood epilepsy (Kuzniecky 1994). Changes in neural progenitor proliferation may result in microcephaly or macrocephaly, both of which have been associated with autism spectrum disorders (ASDs) (Fombonne et al. 1999). The neurodevelopmental analysis of animal models with mutations in ASD risk genes, by others and ourselves, has provided strong evidence that increased neural progenitor proliferation is the mechanism underlying macrocephaly in autism (Golzio et al. 2012; Orosco et al. 2014; Sugathan et al. 2014; Chen et al. 2015; Gompers et al. 2017). While predisposing risk genes for the aforementioned disorders have been recognized over recent years, it is evident that environmental factors may also exert pathological influences on neural progenitor production and cortical neurogenesis. In this context, the essential B_9_ vitamin folic acid (FA) is of particular interest, as animal studies have demonstrated that maternal folate status can modulate fetal neural progenitor generation, having effects on both proliferation and apoptosis (Craciunescu et al. 2004; Craciunescu et al. 2010; Yuan et al. 2013; Sudiwala et al. 2019). In an important public health effort to prevent neural tube defects, FA intake has substantially increased over recent decades through fortification programs as well as the use of FA supplements (Oakley Jr et al. 1995). Since the implementation of a mandatory food fortification program in 1998, FA has been added to enriched flours and other grain products in the United States of America and Canada (Pfeiffer et al. 2005). In addition, dietary FA supplements are available in pure form or as a component in multivitamins. Unsurprisingly, supraphysiologic folate concentrations were found in 23% of the US population, including 43% of children younger than 5 years of age (Pfeiffer et al. 2005). Notably, women who have given birth to children with neural tube defects, and are at increased risk of recurrence, are advised to take 4 mg of FA per day during subsequent pregnancies, 10 times the recommended daily intake (RDI) of 400 μg, considerably exceeding the recommended safe upper limit of 1000 μg daily (1998). In some countries, other populations, such as transfusion-dependent thalassemia patients (Tripathi et al. 2016), patients with hereditary spherocytosis (Paniz et al. 2019), pregnant women with sickle cell disease (Dixit et al. 2016), and women with epilepsy taking anticonvulsants (Morrow et al. 2009) are recommended a daily intake of up to 5 mg of FA, 12.5 times the RDI. Although safe upper limits for folate have been defined (1998), no guidelines have been put in place to prevent excessive consumption from multiple sources of fortified grains, supplemented cereals, and over-the-counter dietary supplements.

The consequences of FA supplementation on neurodevelopmental disorders have been investigated epidemiologically, with a focus on autism incidence rates providing conflicting pictures. Studies have suggested either a protective effect of FA on ASD prevalence (Schmidt et al. 2012; Suren et al. 2013; Schmidt et al. 2019), no association (Virk et al. 2015), or increased risk (Beard et al. 2011; Wiens and DeSoto 2017; Raghavan et al. 2018). Recent data from the Boston Birth Cohort (Raghavan *et al.* 2018) suggest a possible stratification with respect to maternal FA intake and autism incidence by finding that risk was greatest in children born to mothers with the highest maternal plasma folate levels exceeding the cutoff suggested by the WHO (>45.3 nmol/L).

Here, in a mouse model, we describe the effects of maternal FA deficiency or excess on offspring neurodevelopment. We find that FA deficiency or excess comparably alter prenatal neurogenesis leading to cytoarchitectural changes of the cerebral cortex. Biochemical changes suggest that under either condition a relative shift of the folate pathway from DNA methylation to DNA synthesis may be favored. These changes in timing of neurodevelopment and folate metabolism are associated with postnatal behavioral deviations in both FA deficiency and excess groups.

## Materials and Methods

### Animal Husbandry

C57BL/6NJ mice were housed in facilities approved by the Association for Assessment and Accreditation of Laboratory Animal Care International. All animals were handled in accordance with protocols approved by the University of California at Davis Institutional Animal Care and Use Committee. Five-week-old females were supplied with their respective FA controlled diets 2 weeks prior to being placed with a male. Breeding pairs were kept on their respective FA controlled diets until embryo or pup collection. Clifford/Koury-based L-amino acid defined rodent diets with conventional (2 mg/kg), deficient (0 mg/kg), and excess FA (20 mg/kg) content without succinyl sulfathiazole were purchased from Dyets Inc. (Bethelem, PA).

### Tissue Collection and Histological Analysis

Pups were collected at birth [postnatal day (P) 0], and 6 days after birth (P6). They were transcardially perfused at a speed of 1 mL/min (P0) and 2 mL/min (P6) with phosphate-buffered saline (PBS) for 5 min, followed by 4% paraformaldehyde (PFA) in PBS for 10 min. Pups were decapitated and heads further fixed in 4% PFA/PBS overnight. The following day, heads were washed with PBS for 1 h, and then brains removed from skulls. Brains were then transferred to 15% sucrose for 12 h at 4**°**C, then to 30% sucrose for another 12 h at 4**°**C, and finally to OCT compound (Fisher Healthcare) for 45 min at room temperature (RT) to be cryoprotectively frozen. Freezing took place submerged in OCT-filled polyethylene molds set in dry ice-chilled methanol. Embedded brains were stored at −80**°**C until use and sectioned on a Leica CM3050S cryostat, collecting 10 μm sections at every 100 μm on Superfrost Plus glass slides (Thermo Fisher Scientific). Sections were left to dry on a Premiere Slide Warmer XH-2004 overnight and, the following day, kept at −80**°**C until ready to be further processed. Embryo collection took place at embryonic day (E) 14.5. After transcardial perfusion, embryonic brains were collected and fixed in 4% PFA/PBS for 3 h. Subsequently, brains were cryoprotected and then frozen following the previously described steps.

### Immunofluorescence

All immunofluorescence was carried out on slide-mounted sections. In brief, sections were lightly fixed for 10 min at RT in 4% PFA/PBS, then washed in PBS for three times for 5 min each. Heat-induced epitope retrieval was performed using a Biocare Medical Decloaking Chamber NxGen using the DIVA Decloaker solution (Biocare Medical) diluted at 1× working concentration in deionized water. Samples were washed in PBS and incubated with 10% donkey serum in PBS + 0.1% Triton X-100 to permeabilize the tissue and prevent nonspecific antibody binding. Primary antibody was applied and incubated overnight at 4**°**C. The following primary antibodies and respective dilutions were used: Tbr1 (Abcam, ab31940), 1:200; Ctip2 (Abcam, ab18465), 1:200; Brn2 1:200; Pax6 (Covance PRB-278P-100), 1:100; Tbr2 (Abcam, ab183991), 1:100; Ki67 (Abcam, ab15580), 1:200.

### Golgi Staining

From each group, brains of 8 to 10 week-old mice born to different dams were collected. Tissue impregnation was performed using a modified Golgi-Cox method as described in the FD Rapid GolgiStain kit (FD Neurotechnologies). Following impregnation, 100 μm coronal sections were cut on the cryostat and mounted on Superfrost Plus slides for staining with silver nitrate solution. After staining was completed, sections were dehydrated in ethanol and mounted with Permount mounting medium (Thermo Fisher Scientific). Brightfield confocal photomicrographs were acquired using an Olympus microscope with associated software at 40× magnification. Twenty (0 mg/kg FA group), 24 (2 mg/kg FA group), and 22 (20 mg/kg FA group) cells, respectively, were processed for dendritic analysis by acquiring 30–70 μm stacks of 0.34 μm consecutive images and importing those to the Fiji software platform. Neurites were traced following their position along the *z*-axis for optimal accuracy using the “Simple Neurite Tracer” plugin. Reconstruction of somata and dendrites was performed using the ROI manager tool. An arborization analysis was then performed using the Sholl analysis plugin applying a modified Sholl method for best polynomial fit. First and subsequent shells were set at a radius of 1.17 μm and intersections at each Sholl radius were determined. Raw data were compiled and imported onto GraphPad Prism for statistical analysis by mixed model two-way ANOVA followed by post hoc analysis using Tukey’s multiple comparison test.

### EdU Proliferation Analysis of Cortical Neurogenesis

For the 20 h EdU pulse-chase assay, time-pregnant females were injected intraperitoneally with 50 mg/kg bodyweight EdU. After 20 h, females were killed, and embryo tissue processed as described above. Slides were incubated in 2 N HCl at 37°C for 20 min and rinsed two times in PBS with 0.2% Triton X-100. EdU detection was performed after immunostaining according to manufactures Click-it EdU Alexa 594 imaging kit protocol (Life Technologies). The fraction of cells that had exited the cell cycle (Q-fraction) was estimated by counting the numbers of EdU^+^/Ki67^−^ cells basal to the subventricular zone (SVZ) and all EdU^+^ cells in 200 μm cortical segments. Q-fractions were calculated by dividing the number of EdU^+^/Ki67^−^ cells by the total number of EdU^+^ cells. All imaging was carried out on a Nikon Eclipse A1 laser scanning confocal microscope. Merge-channel views were acquired with the associated NIS Elements A1 software.

### RNA In Situ Hybridization

In situ hybridization using a *Gad67* digoxigenin-labeled antisense RNA probe was carried out on 20 μm thick sections following standard protocols and as previously described (Zarbalis and Wurst 2000). In vitro transcription to generate the labeled riboprobe was performed with a *Gad67* containing plasmid obtained from Dr John L.R. Rubenstein (Maddox and Condie 2001).

### Imaging

All light microscopy was conducted on an Olympus BX-DSU microscope and images acquired by Olympus DP71 camera and associated cellSens software (Olympus Corporation). All fluorescent imaging was concluded within 1 week from secondary antibody application to minimize fluorescent signal degradation. Images were taken at 20× magnification for postnatal brains and at 40× magnification for embryonic tissue, using a Nikon A1 confocal microscope and associated NIS Elements software (Nikon Instruments Inc.).

### Counting and Analysis

All histological data were obtained from at least three embryos, pups, or adults of at least two separate litters per nutritional group. Counts were performed in NIS elements by first, measuring cortical length adjusting endpoints according to developmental stage as outlined below. In embryos, the start of the cortex was considered to be the dorsomedial endpoint of the ventricle. A curved line from this point to the end of the ventricle was drawn and considered the entire length of the cortex. At 20%, 50%, and 80% positions of that length 100 μm-wide segments were outlined and cells within these boxes counted. For P0 pups, the start of the neocortex was selected in the same manner as for the embyros. The line was drawn until the end of the neocortex, considered to be where Ctip2 labeled layer V ends, on the ventrolateral aspect of the cortex. After having established neocortical length, 20%, 50%, and 80% positions of that distance from the dorsal endpoint were measured, 200 μm-wide boxes around these points drawn and cells within these segments counted. In P6 pups, the cortical midline was selected as starting point, following the white matter a line drawn until the end of the neocortex, 20%, 50%, and 80% positions identified, and 500 μm-wide boxes set around these points used for counts. Counts of all measures were performed on two neighboring sections of each brain and the numbers averaged to a single data point depicted in the bar diagrams.

### Behavioral Testing

All behavioral testing was performed on juveniles and young adults aged 4–10 weeks. In total, 13 control mice (2 mg/kg FA, 8 males, 5 females), 10 FA deficient mice (0 mg/kg, 6 males, 4 females), and 8 FA excess mice (20 mg/kg FA, 3 males, 5 females) were tested and no overt differences between males and females were observed. Testing was conducted in the following sequence: 1) The “elevated plus maze” indirectly assesses anxiety behavior, as it measures the conflict between exploration of a novel environment and avoidance of brightly lit open areas (Walf and Frye 2007). Time spent on the open arms versus the closed arms provides a quantitative measure of anxiety-related behavior. Each mouse was placed in the center of the cross and allowed to explore the maze for 10 min. The task was recorded by video camera and scored over the recording, by two different scorers, to ensure accuracy. Once a mouse entered the open arms with all four paws time spent in the open was recorded. 2) The “open field test” assesses locomotion and quantifies distance walked in the center versus perimeter to give an indirect measure of anxiety-related behaviors (Seibenhener and Wooten 2015). In a brightly lit open area, mice will tend to stay near the walls of the open field, rather than enter the center region. Habituation to the novelty of the open field, i.e., reduction of time spent in the perimeter and increase in center time, was measured by recording activity in 5-min consecutive intervals over a 15-min period. Testing was performed with two identical open field mouse chambers (San Diego Instruments) and data recorded and processed with accompanying PAS software. 3) “Novel object recognition” constitutes a measure of cognitive ability in the form of memory formation (Antunes and Biala 2012). Mice were habituated for 10 min to two identical objects placed symmetrically in a square chamber. Then, one object was replaced with a novel object of similar shape but different enough to elicit the mouse’s attention. Naturally, mice will spend more time with the novel object compared to the one presented before as it satisfies the animal’s innate exploratory curiosity. Deviations from this general rule may indicate mnemonic cognitive impairment. Testing sessions were recorded by video camera and scored independently by two scorers to ensure accuracy. 4) Sociability was tested in the “three-chambered social approach” (Moy et al. 2004). Gates connecting the three chambers were equipped with laser trackers recording each time the barrier was crossed and amount of time spent in each chamber. Mice tested were placed in the central chamber and allowed to habituate for 5 min by blocking the entrances to the other chambers. Once familiarized, two baskets were placed in each of the chambers, one empty and one containing an unfamiliar sex- and age-matched mouse. Typically, mice will spend a greater amount of time in the chamber containing the other mouse. Any significant deviation in favor of the empty chamber may be indicative of impaired sociability. 5) The “marble bury test” indirectly measures anxiety and/or obsessive compulsive behaviors (Njung'e and Handley 1991; Thomas et al. 2009). Mice were placed in a cage containing 10 marbles in a 2 × 5 layout and allowed to interact with the marbles for 30 min. Subsequently, mice were removed, pictures of the cages taken, and marbles covered by the bedding to at least 50% scored as buried.

### Relative Quantification of Folate-Mediated One-Carbon Metabolism Intermediates by Mass Spectrometry

Brains used for mass spectrometric analysis of folate metabolites were freshly collected from newborn Pups (P0), flash-frozen on dry ice, and stored at −80**°**C until being further processed. An analysis of multiple folates was performed by ultraperformance liquid chromatography–tandem mass spectrometry (UPLC-MS/MS) on six brains of each group derived from three different litters per group as described previously (Pai et al. 2015; Leung et al. 2017). Buffer containing 20 mM ammonium acetate, 0.1% ascorbic acid, 0.1% citric acid, and 100 mM DTT at pH 7.0 was added to brain samples and sonicated for 10 s with a hand-held sonicator at 60% amplitude on ice. Protein was removed by precipitation with addition of two volumes of acetonitrile, mixing for 2 min and centrifugation for 15 min at 12 000 × *g* at 4**°**C. Supernatants were transferred to fresh tubes, lyophilized and stored at −80**°**C prior to the analysis. Lyophilized samples were resuspended in 25 μL deionized water and centrifuged for 5 min at 12 000 × *g* at 4**°**C. Supernatants were transferred to glass vials for UPLC-MS/MS analysis. Metabolites were resolved by reversed-phase chromatography using Acquity UPLC BEH C18 column (50 mm × 2.1 mm; 1.7 μm bead size, Waters Corporation). Solvents for UPLC were the following: Buffer A, 5% methanol, 95% Milli-Q water and 5 mM dimethylhexylamine at pH 8.0; Buffer B, 100% methanol. The column was equilibrated with 95% Buffer A: 5% Buffer B. The sample injection volume was 15 μL. The UPLC protocol consisted of 95% Buffer A, 5% Buffer B for 1 min, followed by a gradient of 5–60% Buffer B over 9 min and then 100% Buffer B for 6 min before re-equilibration for 4 min. The metabolites were eluted at a flow rate of 200 nL/min. The UPLC was coupled to a XEVO-TQS mass spectrometer (Waters Corporation) operating in negative-ion mode using the following settings: capillary 2.5 kV, source temperature 150**°**C, desolvation temperature 600**°**C, cone gas flow rate 150 L/h, and desolvation gas flow rate 1200 L/h. Folates were measured by multiple reaction monitoring (MRM) with optimized cone voltage and collision energy for precursor and product ions. An analysis of peak areas was carried out using MassLynx software (Waters Corporation).

### DNA Purification and Methylation Analysis

Sample global DNA (gDNA) was obtained from ~ 10 mg tissue using Viagen Direct PCR Tail lysis buffer according to manufactures instructions. After lysis, gDNA was purified using phenol:chloroform:isoamyl alcohol (25:24:1; Invitrogen), then precipitated with ethanol and dried. After resuspension in H_2_O, the concentration and purity for gDNA were measured via NanoDrop One^c^ (Thermo Fisher Scientific). All gDNA purity was measured with an OD 260/280 of > 1.6 considered appropriate for the subsequent assay. Methylated cytosine percentages (%5-mC) of 100 ng of gDNA from each sample were measured using the MethylFlash Global DNA Methylation (5-mC) ELISA Easy kit (EpiGentek Group Inc.). The colorimetric absorbance after signal development was measured at 450 nm using a SpectraMax i3X plate reader (Molecular Devices). The %5-mC was calculated based on standard curves (*R*_1_^2^ = 0.978, *R*_2_^2^ = 0.986) established following the manufacturer’s instructions.

### Western Blotting and Analysis

Brain and liver tissue were collected, flash frozen on dry ice, and lysed in RIPA lysis buffer. Protein concentration in individual samples was determined by bicinchoninic acid protein assay (Pierce). Of each lysate, 10 μg of protein per well were loaded into Novex Tris-Glycine Wedgewell polyacrylamide gels (Thermo Fisher Scientific). After electrophoresis and transfer to nitrocellulose membranes, 4% milk protein in Tris-buffered saline (TBS) with 0.1% Tween-20 (TBST) was applied for 1 h at RT. Membranes were then incubated overnight at 4**°**C with Mthfr (1:1500; Milipore) and Vcl (1:1500; Cell Signaling Technology) antibodies diluted in TBST/1% milk. Subsequently, secondary α-rabbit horseradish peroxidase-conjugated antibodies were applied for 1.5 h at RT (Jackson ImmunoResearch). The membranes were then incubated with enhanced chemiluminescence substrate (Thermo Fisher Scientific) and imaged on a Bio-Rad ChemiDoc Imager (Bio-Rad Laboratories, Inc.). Quantifications were performed by establishing relative signal intensity ratios of Mthfr over vinculin (Vcl) loading control using Bio-Rad Image Lab software. Brain lysates exhibited additional bands compared to liver samples. For brain, we used Mthfr (71 kDa) and p-Mthfr bands for relative signal intensity measurements and for liver Mthfr (71 and 77 kDa) and p-Mthfr bands.

### Statistics

All statistical analyses were performed with Graphpad Prism 6.0e. Initially, datasets were cleaned using the ROUT outlier test with the Q value set to 1%. Subsequently, variance, standard deviation, and normality (Shapiro–Wilk) were tested when appropriate to analyze datasets. All statistical tests, aside from DNA methylation, were parametric, unpaired Student’s *t*-tests independently comparing the two FA treatment groups to control animals receiving a conventional FA diet. Results were considered to be statistically significant if *P* ≤ 0.05. In the figures, bar diagrams depict the mean and standard deviation above and below the mean. Individual data points correspond to biological replicates of one embryo, pup, or adult, respectively. The extent of significance between groups is indicated with one, two, three, or four asterisks if *P*-values were equal to or less than 0.05, 0.01, 0.001, or 0.0001, respectively. DNA methylation data were tested with a standard Mann–Whitney nonparametric unpaired *t*-test.

## Results

### FA Deficiency or Excess Alters Cortical Morphology and Neuron Populations at Postnatal Stages

To test the effects of gestational maternal FA intake on the offspring, we generated three test groups of C57BL/6NJ dams that were fed amino acid defined rodent chow (Dyets Inc.) containing no FA (deficient), 2 mg (control), or 20 mg of FA per kg chow (excess). Diets were initiated 2 weeks prior to breeding and maintained throughout pregnancy. Pups were collected on P0 and P6 and brains processed for histological analysis. We focused our investigation on the cerebral cortex, in humans the primary site of higher-order functions, such as cognitive and emotional processing and central to a range of psychiatric disorders, particularly those that first appear during childhood (Krystal and State 2014).

**Figure 1:**
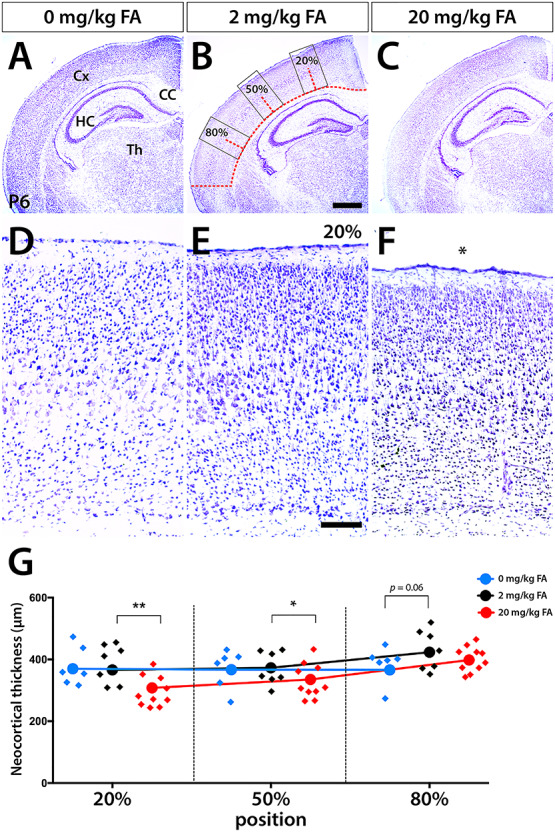
Cortical morphology at different levels of FA supplementation in mice. Nissl-stained P6 forebrain sections reveal changes in dorsomedial cortical thickness under conditions of FA deficiency (0 mg FA/kg chow) (*A*, *D*, blue data in *G*) and FA excess (20 mg/kg chow) (*C*, *F*, red data in *G*) compared with controls (2 mg/kg chow) (*B*, *E*, black data in *G*). CC, corpus callosum; Cx, cortex; HC, hippocampus; Th, thalamus. Scale bar is 1 mm in (*B*) and 100 μm in (*E*).

First, we examined cortical morphology at the mesoscale by analyzing Nissl stained forebrain sections at P6. Overt differences in neocortical length were not observed between test groups. To accurately detect possible differences in cortical thickness, we measured neocortical length of select matched representative sections of each brain, identified 20% (dorsal), 50% (mediolateral), and 80% (lateral) distances from the dorsal midline and measured cortical thickness at these positions. Measurements of cortical thickness at the preidentified positions showed a notable thinning of the cerebral cortex in the excess folate group (20 mg/kg FA). Neocortical thinning was not uniform, with greatest effect seen at dorsal aspects (20% position), where we identified an ~ 16% thinner cortex compared with controls (*P* = 0.003). At mediolateral aspects (50% position), a significantly ~ 10% thinner cortex was observed (*P* = 0.023), and no significant difference at the lateral 80% position was identified (Fig. 1). The FA deficient group displayed only a trend toward thinning at lateral aspects (80% position) with no other significant difference observed [366.6 ± 40.2 μm (0 mg/kg), 423.7 ± 55.4 μm (2 mg/kg), *P* = 0.061).

**Figure 2:**
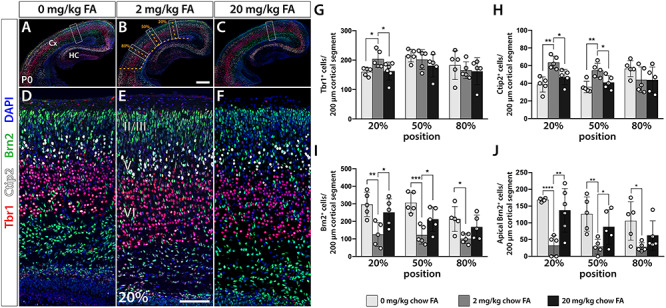
Maternal FA intake during gestation alters cortical projection neuron numbers in the offspring. (*A–F*) Immunofluorescent analysis of cortical layer markers Tbr1 (layer VI), Ctip2 (layer V), and Brn2 (layers II, III, and V) in the brains of newborns (P0) reveals significantly reduced numbers of early-born neurons (Tbr1^+^ and Ctip2^+^) under conditions of FA deficiency (0 mg/kg chow) and FA excess (20 mg/kg chow) compared with controls (2 mg/kg chow) (*G*, *H*). In contrast, the number of late-born Brn2^+^ cells is substantially increased (*I*, *J*). This increase affects particularly cells apical to the cortical plate (*J*), the migration of which appears delayed in the dietary extremes. Regional loss of Tbr1^+^ and Ctip2^+^ cells confirms that dorsomedial cortical positions (20%) are more strongly affected than ventrolateral positions (*G*, *H*). Significant differences are indicated by asterisks. Scale bar in (*B*) is 250 μm and in (*E*) 50 μm.

To assess which cell types may contribute to morphological differences associated with varying gestational FA supply, we opted to examine cortical projection neurons, the predominant neuron population in the cortex at both P0 and P6. To do so, we applied immunofluorescent analysis using layer-specific markers Tbr1 (layer VI), Ctip2 (layer V), and Brn2 (predominantly layers II and III) (Kwan et al. 2012) to both quantify projection neuron subtypes and identify disturbances in layer organization. To portray a representative picture of cortical cell populations, we measured neocortical length to locate 20%, 50%, and 80% distances from the dorsal midline of each brain and performed cell counts in segments stretching 100 μm at P0 or 250 μm at P6 in either direction of these distance marks. No overt alterations in layer organization between test groups were observed, but we noticed differences in the numbers of each cell type analyzed. Specifically, at P0 we found that changes seen with excessive maternal FA intake mirror those seen in FA deficiency, with reduced numbers of early-born neurons (Tbr1, Ctip2), but increased numbers of late-born neurons (Brn2) (Fig. 2). Intriguingly, this change in neocortical cellular composition was distinctly regional with mostly dorsal (20%) aspects being significantly affected in test groups compared with control (2 mg/kg) [Tbr1, 160.2 ± 13.9 (0 mg/kg) *P* = 0.016, 204.0 ± 29.1 (2 mg/kg), 163.3 ± 31.0 (20 mg/kg) *P* = 0.049; Ctip2, 38.6 ± 8.6 (0 mg/kg) *P* = 0.001, 63.6 ± 7.2 (2 mg/kg), 45.2 ± 8.8 (20 mg/kg) *P* = 0.005; Brn2, 296.6 ± 69.4 (0 mg/kg) *P* = 0.004, 125.6 ± 68.1 (2 mg/kg), 274.5 ± 89.6 (20 mg/kg) *P* = 0.014]. Notably, the number of late-born Brn2^+^ neurons located apically to layer VI was significantly increased in both deficient and excess FA test groups across most cortical positions (except for the lateral, 80% position of the 20 mg/kg group) with the 20% site most affected [20%, 168.5 ± 5.8 (0 mg/kg) *P* < 0.0001, 32.0 ± 29.8 (2 mg/kg), 137.2 ± 65.1 (20 mg/kg) *P* < 0.0001; 50%, 126.0 ± 51.2 (0 mg/kg) *P* = 0.0045, 28.6 ± 21.9 (2 mg/kg), 87.8 ± 51.5 (20 mg/kg) *P =* 0.046; 80% 105.6 ± 57.5 (0 mg/kg) *P* = 0.018, 27.4 ± 13.1 (2 mg/kg), 62.0 ± 44.1 (20 mg/kg) *P =* 0.13], suggesting a delay in the generation and migration of late-born neurons (Fig. 2*D*–*F*, *J*). We followed up this observation by assessing the same projection neuron populations in brains collected at P6, a time point at which developmental neurogenesis, neuronal migration, and gliogenesis should largely have been concluded. Again, we found a dorsally restricted loss of early-born neurons [20% position, Tbr1, 464.7 ± 68.0 (0 mg/kg) *P* = 0.004, 786.3 ± 59.2 (2 mg/kg), 531.6 ± 38.4 (20 mg/kg) *P* = 0.0003; Ctip2, 179.7 ± 22.3 (0 mg/kg) *P* = 0.031, 263.0 ± 38.2 (2 mg/kg), 201.8 ± 24.6 (20 mg/kg) *P* = 0.031], but increase in Brn2^+^ late-born-neurons [20% position, 211.6 ± 54.3 (0 mg/kg) *P* = 0.016, 133.4 ± 19.1 (2 mg/kg), 240.8 ± 84.2 (20 mg/kg) *P* = 0.026] in both groups at the FA dietary extreme (Supplementary Fig. 1). At P6, Brn2^+^ cells had completed their migration and ascended to their basal locations in both FA test and control mice. However, particularly in the FA excess group small numbers of Brn2^+^ could still be observed in deeper neocortical layers (Supplementary Fig. 1*F*). In summary, our results confirmed a persistent and comparable deviation in cortical cytoarchitectural organization under conditions of both FA deficiency and excess.

**Figure 3:**
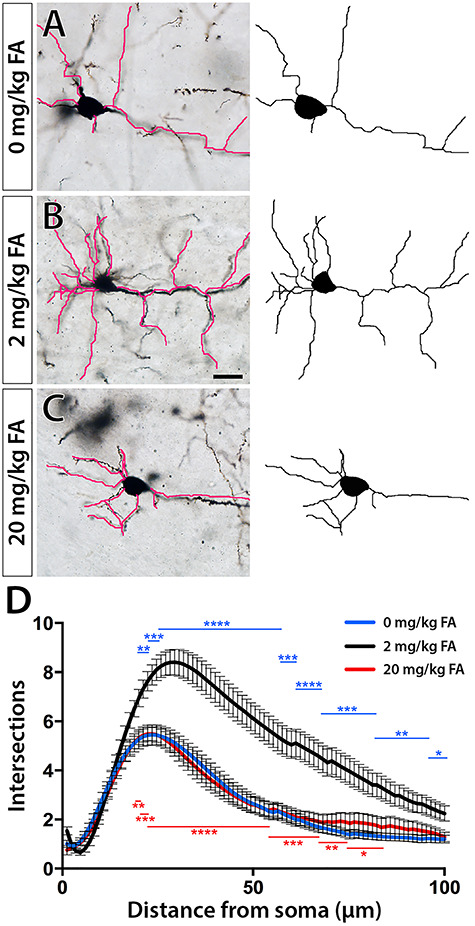
Sholl analysis of Golgi-stained projection neurons reveals differences between FA test groups and controls. (*A*–*C*) Representative micrographs and tracings illustrate reduced dendritic arborization in FA deficiency (*A*, 0 mg/kg chow) and excess (C, 20 mg/kg chow) compared with control neurons (*B*, 2 mg/kg chow). (*D*) Sholl profiles confirm significant differences between both test groups and controls. Significant differences are indicated by blue and red asterisks for FA deficient and excess neurons respectively. Scale bar is 20 μm.

The observation of persistent changes in neocortical cellular composition prompted us to examine whether these shifts have lasting effects on cortical circuitry and cellular morphology. The complexity of dendritic arbors can be a proxy for neural circuit complexity and significant changes may be associated with neuronal dysfunction (Cline 2001; Parrish et al. 2007; Jan and Jan 2010). To that end, we collected brains from adult offspring (~6–8 weeks old) of control and FA dietary test groups and performed Golgi staining to assess dendritic arborization using Sholl analysis. We focused our analysis on deep-layer projection neurons of somatosensory cortex at bregma −1.5 to −1.8 mm around the 20% position, the population of cells that our previous assessments confirmed as numerically most diminished. Intriguingly, despite some heterogeneity of cortical projection neurons in all groups, we found Sholl profiles to be significantly almost identically reduced in complexity among neurons of FA deficient and excess offspring compared with control neurons (Fig. 3; *P* ≤ 0.05–0.0001 between test group and control from 17 to 82 μm distance from the soma). Our results indicated overlapping changes in cortical circuitry between FA deficiency and excess.

Considering our observations of changes in projection neuron types, we sought to investigate whether neocortical interneurons are subject to alterations as well. Performing *Gad67* RNA in situ hybridization on P6 brains and focusing our analysis on the 20% neocortical position, previously established as the site of greatest effects, we did not identify variations in interneuron numbers and distribution associated with FA availability [308.0 ± 28.5 (0 mg/kg) *P* = 0.57, 278.8 ± 43.6 (2 mg/kg), 360.6 ± 59.3 (20 mg/kg) *P* = 0.36] (Supplementary Fig. 2).

### FA Deficiency and Excess Alters Prenatal Cortical Neurogenesis

**Figure 4:**
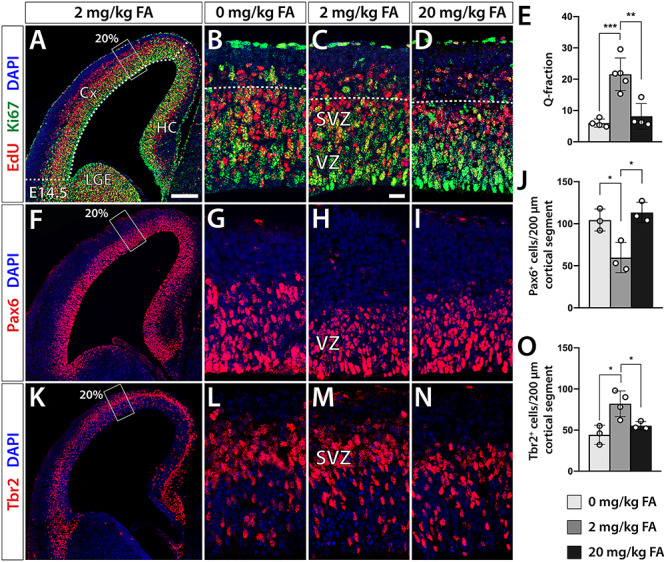
Prenatal neurogenesis is altered in FA experimental groups at peak neurogenesis. Analysis at E14.5 uncovers shifts in neural proliferation of the FA dietary groups at the extremes that mirror each other. (*A–D*) EdU/Ki67 analysis reveals a decrease in neuron generation in FA deficiency (0 mg/kg) and excess (20 mg/kg) as demonstrated by a reduced quit (Q-) fraction in both groups, when compared with controls (2 mg/kg) (*E*). (*F–I*) Immunofluorescence analysis of Pax6^+^ cells indicates a significant increase in RGCs of the two FA test groups (*J*). (*K–N*) The Tbr2^+^ IP population is significantly decreased in both FA experimental groups compared with controls (*O*). SVZ, subventricular zone; VZ, ventricular zone. Significant changes are indicated by asterisks. Scale bar in (*A*) is 200 μm and in (*C*) 20 μm.

In the mouse, cortical projection neurons are born during E11.5–18.5 (Farkas et al. 2008; Martynoga et al. 2012), suggesting that the morphological abnormalities that we observed in our FA test groups may be the consequence of altered prenatal proliferative dynamics. Consequently, we opted to test the rate of neuron generation at E13.5–14.5, a period of peak neurogenesis and birth of early-born neurons in the mouse. To that end, we employed a 20 h pulse-chase thymidine labeling approach using 5-ethynyl-2′-deoxyuridine (EdU) in brain sections to measure the quit (Q)-fraction of cells that have excited the cell cycle over the labeling period. To label and exclude cells still undergoing division, we used the proliferation marker Ki67. Taking into consideration the regional effects of FA manipulation, we focused exclusively on the 20% position where we counted the number of EdU^+^/Ki67^−^ cells basal to the SVZ and total number of EdU^+^ cells in 200 μm wide cortical segments. Establishing the ratio between these two cell populations, we found a significant ~ 72% reduction of the Q-fraction (Student’s *t*-test, *P* < 0.0005) in FA deficient and ~ 57% reduction in FA excess groups respectively [0.06 ± 0.01 (0 mg/kg) *P* < 0.001, 0.22 ± 0.05 (2 mg/kg), 0.09 ± 0.04 (20 mg/kg) *P* = 0.006] (Fig. 4*A*–*E*). Both results were consistent with the loss of early-born neurons observed at postnatal stages and prompted us to examine whether this reduction in neurogenic rates is accompanied by specific changes in the cellular composition of the cortical proliferative compartments as well. Analyzing radial glial cells (RGCs) and intermediate progenitors (IPs), by applying Pax6 and Tbr2 immunofluorescence, respectively, we identified at the 20% position significant 75% (0 mg/kg) and 95% increases (20 mg/kg) in the number of Pax6^+^ cells, respectively, compared with controls [104.3 ± 13.1 (0 mg/kg) *P* = 0.025, 59.7 ± 18.0 (2 mg/kg), 113.3 ± 12.1 (20 mg/kg) *P* = 0.013] (Fig. 4*F*–*J*) and significantly reduced numbers of Tbr2^+^ cells [44.3 ± 11.6 (0 mg/kg) *P* = 0.018, 82.0 ± 15.7 (2 mg/kg), 55.3 ± 4.9 (20 mg/kg), *P* = 0.039] (Fig. 4*K*–*O*). In line, with the findings described above of a delay in neurogenesis associated with maternal FA supply at the extremes, the bottleneck in neuronal differentiation appears to occur at the RGC stage preventing timely transition to IP.

### Increased Apoptotic Rates in FA Deficient and FA Excess Offspring

**Figure 5:**
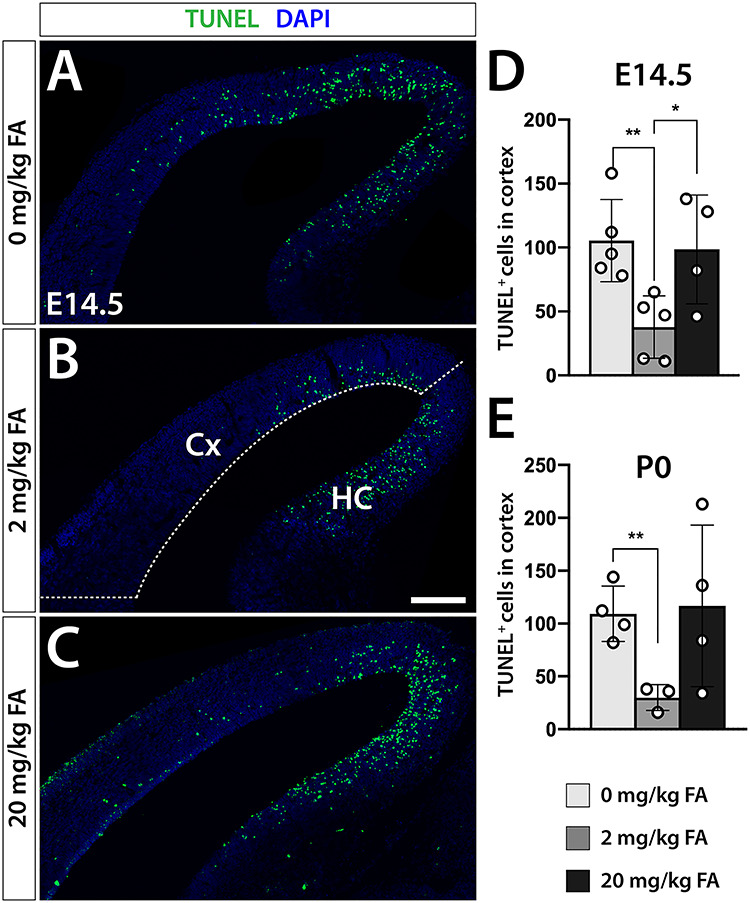
FA deficiency or excess increase apoptotic rates in the developing cortex. TUNEL analysis reveals a significant increase in TUNEL^+^ cells under conditions of FA deficiency (*A*, 0 mg/kg chow) or excess (*C*, 20 mg/kg chow) compared with controls (*B*, 2 mg/kg chow), at both E14.5 (*D*) and birth (P0, *E*). Cx, cortex; HC, hippocampus. Scale bar is 250 μm.

To examine whether differences in projection neuron distribution in FA test groups were exclusively the consequence of altered proliferative dynamics of neural progenitors, we examined apoptotic rates using TUNEL assay in brain sections of E14.5, P0, and P6 offspring. Interestingly, at stages E14.5 and P0, we detected significantly increased apoptosis in the developing cortex of both FA deficiency and excess groups in keeping with the parabolic pattern of neuronal developmental changes noted before. Specifically, at E14.5, we recorded a significant ~ 2.5-fold increase in apoptotic rates in the FA deficient and excess groups compared with controls [105.4 ± 32.1 (0 mg/kg) *P* = 0.011, 46.2 ± 30.0 (2 mg/kg) 98.5 ± 42.7 (20 mg/kg), *P* = 0.05] (Fig. 5*A*–*D*). Similarly, at P0, we found a ~3-fold increase in cells undergoing programmed cell death in the deficient group [109.3 ± 26.2 (0 mg/kg), *P* = 0.005, 30.0 ± 12.2 (2 mg/kg)] and a comparable trend in the 20 mg/kg group that did not reach statistical significance (116.8 ± 76.5 *P* = 0.12) (Fig. 5*E*). In all groups, regardless of FA status most apoptotic cells were concentrated in dorsal neocortical aspects at E14.5 and P0 providing an additional explanation as to why this region appears most affected by the loss of some neuron types in FA deficiency and excess. Irrespective of FA status, at P6 apoptotic rates within the excess group had decreased substantially while the deficient group's rates were slightly lower compared with controls [22.33 ± 11.02 (0 mg/kg) *P* = 0.19, 36.33 ± 11.02 (2 mg/kg), 15.20 ± 4.97 (20 mg/kg) *P* = 0.009] (Supplementary Fig. 3).

### Changes in Folate Species Distribution in Brains of FA Deficient and Excess Pups

**Figure 6:**
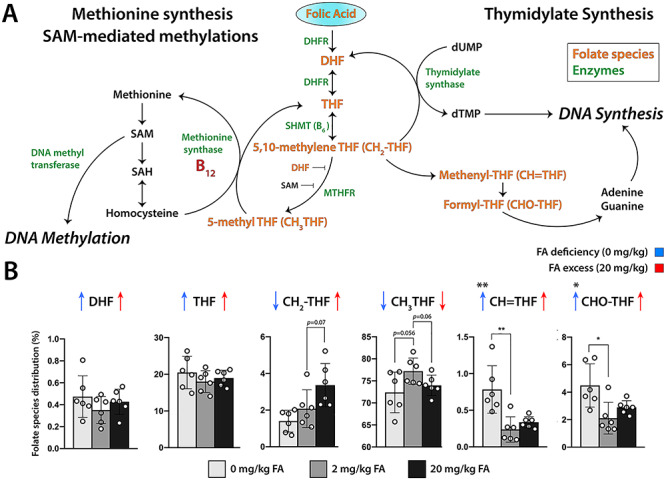
Folate pathway dysregulations in offspring gestated under different FA maternal intake conditions. (*A*) Diagram illustrating the folate pathway and its relationship to DNA synthesis and DNA methylation [modified from (Crider et al. 2012)]. (*B*) LC–MS/MS measured folate species distribution in brains of newborn pups (P0) gestated under control, (2 mg/kg), folate deficiency (0 mg/kg), and excess (20 mg/kg) maternal intake conditions. Significant changes are indicated by asterisks. The *P*-values of borderline nonsignificant differences between test groups are indicated as well. DHF, dihydrofolate; Dhfr, dihydrofolate reductase; Mthfr, methylenetetrahydrofolate reductase; Shmt, serine hydroxymethyltreansferase; THF, tetrahydrofolate.

To investigate whether maternal dietary FA affected folate metabolism as reflected in folate species distribution in test groups, we collected brains at P0 and performed mass spectrometry-based analysis on hemisected brains to determine the folate profile of the six major folate species and their polyglutamated forms (N 1–7). We found shifts in folate profiles in brains of mice exposed to both prenatal FA deficiency and excess. Interestingly, with the exception of methylenetetrahydrofolate (CH_2_-THF) that showed a trend toward increase with increasing maternal FA intake, all other folate species displayed trends in the same direction in either treatment group. Specifically, we found significant relative increases of methenyltetrahydrofolate [CH = THF, 0.78 ± 0.32% (0 mg/kg), 0.24 ± 0.17% (2 mg/kg), *P* = 0.004] and formyl-THF (CHO-THF, 4.48 ± 1.6% (0 mg/kg), 2.11 ± 1.2% (2 mg/kg), *P* = 0.013) in FA deficient offspring compared with controls and trends in the same direction in excess group animals (Fig. 6). This finding suggested a diversion of CH_2_-THF toward CH = THF and CHO-THF rather than conversion through methylenetetrahydrofolate reductase (Mthfr) to methyl-THF (CH_3_-THF). CH = THF and CHO-THF are required for both pyrimidine (thymidine) and purine DNA substrate biosynthesis in contrast to CH_3_-THF that serves as the primary methyl donor for subsequent methylation reactions, including DNA methylation. Indeed, relative CH_3_-THF abundance showed borderline significant trends toward decreases in either FA deficient [72.38 ± 4.6% (0 mg/kg), 77.2% ± 3.0% (2 mg/kg), *P* = 0.057] or FA excess offspring (73.99 ± 2.3%, *P* = 0.063), suggesting that neither of these conditions, deficiency or excess, favor diversion of one-carbon groups toward methylation. In the FA excess groups, this is also consistent with reported inhibition of Mthfr by high doses of FA (Christensen et al. 2015).

Based on these findings, we decided to examine whether the observed redistribution of one-carbon groups in FA directed folates toward nucleotide synthesis at the possible expense of transfer to the methylation cycle via CH_3_-THF had any effects on global DNA methylation. Using purified DNA from P0 brains and livers of each dietary group, we measured mean methylated cytosine percentages (%5-mC) using the MethylFlash Global DNA Methylation (5-mC) ELISA Easy kit (EpiGentek Group Inc.). While we observed a significant ~ 3-fold increase in the proportion of methylated liver DNA in the FA excess group this effect was not seen in livers of the FA deficient group [0.37 ± 0.26 (0 mg/kg) *P* = 0.28, 0.24 ± 0.13 (2 mg/kg), 0.74 ± 0.37 (20 mg/kg) *P* = 0.01] (Supplementary Fig. 4). Further, no significant differences were observed in global DNA methylation states in the brains of either FA deficient or excess offspring.

Finally, we examined whether Mthfr levels were dysregulated in either FA test group compared to control by performing western analysis on protein lysates obtained from liver and brain at P0. The analysis of either tissue did not show any significant differences in expression of phosphorylated or nonphosphorylated Mthfr between offspring groups (Supplementary Fig. 5).

### Behavioral Alterations in FA Deficient and FA Excess Offspring

**Figure 7:**
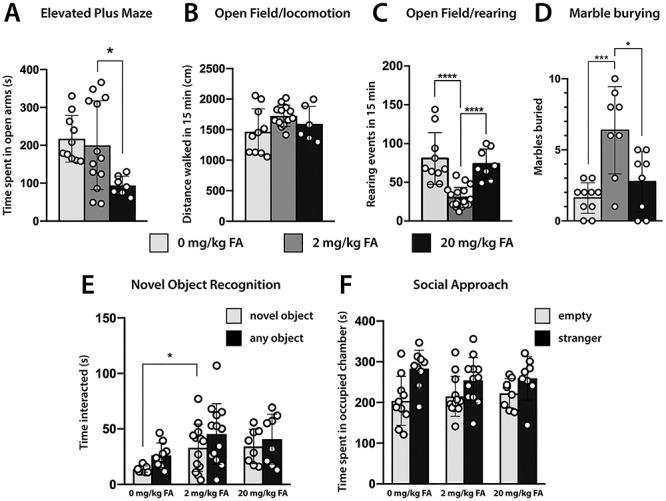
Behavioral abnormalities in young adult offspring gestated with deficient or excess FA supply. (*A*) In the elevated plus maze test, offspring of FA excess supplied dams significantly avoided the open arms of the maze, suggesting heightened anxiety. This is not associated with decreased motility, as all groups performed comparably in open field exploration (*B*). (*C*) Unsupported rearing frequency was significantly increased in both test groups compared with controls. (*D*) In the marble burying test both groups at the dietary extremes buried fewer marbles compared with controls. (*E*) Novel object recognition identified a significant underperformance in FA deficient offspring compared with controls, but no changes in FA excess mice. (*F*) Social approach revealed no differences between FA test group and controls.

To test whether the cortical morphological abnormalities elicited by conditions of gestational FA deficiency and excess affect behavior of the offspring, we tested young mice (4–6 weeks old) using behavioral assays in the following order: elevated plus maze, open field, novel object recognition, 3-chambered social approach, and marble burying (Fig. 6). In the elevated plus maze, a test designed to examine anxiety-related behaviors in rodents (Walf and Frye 2007), we found that offspring of dams fed an excess of FA (20 mg/kg) spent significantly less time (~50%) in the open arms compared to the folate control group [200.1 ± 117.3 s (2 mg/kg), 93.7 ± 24.9 s (20 mg/kg), *P =* 0.03] (Fig. 7*A*). Mice born to folate-deficient dams (0 mg/kg) did not exhibit differences in time spent in open versus closed arms (217.3 ± 61.6 s, *P* = 0.68). Testing the same mice in the open field, revealed less distance traveled for the FA deficient group, but no significant differences in walking distance of FA excess offspring, confirming that less time spent in the open arms of the elevated plus maze by this group was not an effect of impaired motility [1465.0 ± 375.2 cm (0 mg/kg) *P* = 0.036, 1726.0 ± 173.0 cm (2 mg/kg), 1593.0 ± 290.7 cm (20 mg/kg) *P* = 0.225] (Fig. 7*B*). Interestingly, unsupported rearing frequency in the open field, a proxy for exploratory, anxiety, and repetitive behavior (Ennaceur 2014) was significantly increased in both deficient and excess FA groups compared with control animals [rearing events in 15 min were 80.7 ± 33.3 (0 mg/kg) *P* < 0.0001, 30.2 ± 13.2 (2 mg/kg), 73.5 ± 19.5 (20 mg/kg) *P* < 0.0001] (Fig. 7*C*). To further examine repetitive and/or anxiety-related behaviors, we performed the marble burying test. In this test, both FA deficient and excess groups buried significantly fewer marbles (out of 10 in the cage) in a 30-min window [buried marbles average: 1.6 ± 1.1 (0 mg/kg) *P* < 0.001, 6.4 ± 3.1 (2 mg/kg), 2.8 ± 2.1 (20 mg/kg) *P* = 0.016] (Fig. 7*D*). We then proceeded to assess short-term memory of test mice by their ability to discriminate familiar from novel objects (Antunes and Biala 2012). We found that mice born to FA-deficient dams significantly underperformed in this test, not only spending less time with the novel object than the control group [14.3 ± 3.0 s (0 mg/kg), 29.4 ± 10.3 s (2 mg/kg), *P* = 0.003], but also exhibiting decreased interest in the familiar object as well (Fig. 7*E*). The FA excess group did not show any significant deviation from the control group, with respect to time spent with the novel object or total time spent with any object. In the 3-chambered social approach test (Moy et al. 2004), mice from either test group did not exhibit any difference compared with controls, suggesting that mice 4–6 weeks old may not be affected in their social interactions. (Fig. 7*F*) In summary, our results confirm behavioral deviations of test mice born to FA deficient and excess dams with respect to anxiety and memory. While rearing events in the open field and marble burying point to commonalities between the FA experimental groups both elevated plus maze and novel object recognition distinguishes FA deficient from excess offspring.

## Discussion

In the cerebral neocortex, the site of higher-order capacities of the human brain, six horizontal layers can be distinguished, each populated by excitatory and inhibitory neurons of distinct identities and functions (Gupta et al. 2002; Wonders and Anderson 2006; Kwan *et al.* 2012; DeFelipe et al. 2013). Excitatory neocortical projection neurons originate prenatally in the proliferative ventricular and SVZs that contain RGCs and IPs, respectively. These progenitor populations produce neuronal diversity through a temporally tightly regulated series of symmetric and asymmetric divisions (Gotz and Huttner 2005). Specification of projection neuron type is determined by time point and order of birth and correct laminar positioning attained by migration along radial glial processes. While this entire process is genetically regulated, environmental exposures, such as alcohol (Ikonomidou et al. 2000), hypoxia (Vasilev et al. 2016; Morton et al. 2017), toxins (Mousa et al. 2018), or maternal inflammation (Choi et al. 2016), may exert influences that can alter prenatal neurogenesis potentially producing pathological outcomes.

Here we show that maternal FA status during pregnancy can cause widespread changes in cortical neuron generation by altering the numerical relationship between early-born deep layer and late-born upper layer neurons. Specifically, we recognize that both FA deficiency and excess during pregnancy comparably create an excess in late-born Brn2^+^ upper layer neurons, likely at the expense of early-born deep layer Tbr1^+^ and Ctip2^+^ neurons. This effect is highly regional with predominantly dorsomedial cortical aspects affected. The origins of this phenomenon lie in an apparent delay in the transition of Pax6^+^ RGCs to Tbr2^+^ IPs during early neurogenesis, depressing neurogenic rates, as analysis at E14.5 confirmed. Decreased deep layer neuron generation is further exacerbated by increased apoptosis in both FA deficiency and excess. Our findings of changes in cortical cytoarchitecture are in line with observations made in multiple mouse models of neurodevelopmental disorders including mice for 16p11.2 deletion syndrome (Pucilowska et al. 2015), *Cntnap2^−/−^* mutation (Penagarikano et al. 2011), *Fmr1^−/−^* mutation (Lee et al. 2019), and maternal immune activation via the interleukin 17A pathway (Choi *et al.* 2016). Changes in the cellular composition of the neocortex in both FA deficient and excess groups apparently have consequences for cellular morphology and connectivity as well, as Sholl analysis of Golgi-stained projection neurons revealed. Indeed, projection neurons of both FA deficient and excess offspring show diminished complexity and arborization compared with controls suggesting again striking parallels between the two dietary interventions. These findings also fit in with studies that have reported dendritic abnormalities in several human neurodevelopmental syndromes associated with intellectual disability such as Down, Rett, and fragile-X syndromes (Kaufmann and Moser 2000).

Altered neurogenesis arising from folate availability apparently does not extend to cortical interneurons, as their numbers appeared comparable across FA groups. This may be the consequence of their different mode of generation within subpallial domains and without the strict birth sequence distinguishing progenitors and postmitotic subtypes that characterizes projection neurons (Anderson et al. 1997; Lavdas et al. 1999; Anderson et al. 2001; Mi et al. 2018). However, our analysis did not extend into evaluating distinct interneuron subtypes, which may undergo numerical shifts as well, following the paradigm set by the projection neurons. Assuming that extremes in FA supply can change prenatal neurogenesis in humans and nonhuman primates as well, it would be of interest to know to what extent interneurons may be affected, as in these instances a considerable proportion of cortical interneurons is born in the cortical VZ/SVZ (Letinic et al. 2002; Petanjek et al. 2009; Radonjic et al. 2014). Such detailed analysis will be the focus of future studies, as well as whether other brain structures outside the neocortex are subject to comparable cytoarchitectural changes resulting from variations in FA supply.

Mass spectrometric analysis of folate species confirmed changes in the folate profile of test animals compared with controls. Intriguingly, parallels also emerged here between FA deficiency and excess. We observed relative borderline significant reductions in CH_3_-THF in both FA deficient and excess pups compared with controls, a finding that corresponds well with observations made in patients with cerebral folate deficiency (CFD), where reduced CH_3_-THF values present a recurring feature (Ramaekers and Blau 2004). Diminished CH_3_-THF levels are also consistent with reported inhibition of Mthfr by high dose FA (Christensen et al. 2015). Significant relative increases were observed for CH = THF and CHO-THF in the FA deficient group, suggesting a diversion of the folate pathway toward purine and thymidylate synthesis, a rate-limiting step in DNA biosynthesis and cell proliferation (Crider et al. 2012). While FA excess offspring did not show significant relative increases in CH = THF and CHO-THF, rising trends were observed here as well. We propose that this interesting parabolic effect of FA may arise from the fundamental difference in the metabolism of synthetic FA versus naturally occurring reduced folates, as FA must first be reduced to DHF and then to THF before it can enter the functionally active folate pool. Both reactions require the enzyme dihydrofolate reductase (Dhfr) while subsequent conversion of THF to CH_3_-THF requires the enzyme Mthfr. Excess FA may overwhelm the available capacity of either enzyme, producing folate pathway dysregulations comparable to those of folate deficiency. Indeed, such effects have been demonstrated for Mthfr that can be inhibited by high levels of FA, producing pseudo-Mthfr deficiency (Christensen et al. 2015). In support for this concept, Mthfr levels were not significantly changed in brains or livers of newborns born to dams that had received excess FA. The shift of the folate pathway toward thymidylate synthesis in FA test groups may come at the expense of available methyl groups, a possibility we sought to test by assessing global DNA methylation. Our results did not confirm deviations in the proportion of methylated DNA in FA test groups. This may be explained by the maternal supply of alternative methyl group donors, e.g., methionine, betaine, or choline, provided in utero (Pauwels et al. 2017). These sources appear sufficient to compensate for CH_3_-THF loss even in *Mthfr^−/−^* null embryos as to not affect overall DNA methylation (Leung et al. 2017). Similar observations were made in embryos of dams fed a FA deficient diet (Burren et al. 2008). However, dietary manipulation of folate supply, whether direct or indirect, may still exert site specific as opposed to global effects on DNA methylation. Intriguingly, such changes in DNA methylation were recently reported in candidate human loci by comparing continued FA supplementation through trimesters 2 and 3 of pregnancy versus no FA supplementation using cord blood as source material (Caffrey et al. 2018). Separately, it was reported that low maternal folate reduces *Tp53* methylation in adult offspring (McKay et al. 2011).

Behavioral abnormalities associated with variations in gestational folate supply were previously reported in other studies. A CFD rat model with reduced fetal brain folate availability displayed ASD relevant communication, learning, and cognitive deficits (Desai et al. 2017). In a separate study, feeding dams a high FA containing diet at 20 mg/kg chow, pseudo-Mthfr deficiency was observed in the offspring and underperformance in novel object recognition was noted (Bahous et al. 2017). Interestingly, this short-term memory impairment was associated with reduced hippocampal volumes. While our experiments did not replicate these findings, study designs differed considerably. Bahous et al. initiated FA excess in prospective dams at weaning (3 weeks of age) while we induced FA excess in adulthood prior to mating. Behavioral changes in the FA test groups reported in the present study manifest along multiple domains. Mice born to FA deficient dams underperform in novel object recognition, suggesting cognitive defects while mice born to FA excess dams show an anxiety-related phenotype in the plus maze test by significantly avoiding the open arms of the maze. Interpretation of the marble burying test is quite controversial in its having been associated with anxiety-related (Njung'e and Handley 1991) and obsessive compulsive behaviors (Thomas et al. 2009). Both FA test groups buried a smaller number of marbles than controls, excluding increased compulsive behavior, and also did not show signs of decreased locomotive or exploratory behavior in the open field test. This suggests that marble burying in our FA deficiency and excess models may reflect some form of anxiety toward marbles being considered harmful and therefore avoided. This notion may correlate well with increased unsupported rearing in the open field, a behavior that can be associated with increased anxiety (Ennaceur 2014), and greater avoidance of the open arms in the elevated plus maze by the FA excess group.

## Notes

We thank Juliano Bertinato, Abigail Mende, Madeleine Nate, Michael Podesta, Sarah Teel, Irais Baez, Avni Shah, Madhav Sharma, and Albert Lee for technical assistance. N.G. and K.Y.L. are grateful for support from the UCL Great Ormond Street Hospital Biological Mass Spectrometry Centre and Biomedical Research Centre. *Conflict of Interest*: None declared.

## Data and materials availability

All data are available in the main text or the supplementary materials.

## Funding

Elissa Leonard, Powell Family Charitable Trust (R.G.); Shriners Hospitals for Children (K.S.Z.); UC Davis Department of Pathology and Laboratory Medicine (K.S.Z.); UC Davis MIND Institute (K.S.Z.); National Institute of Mental Health (R21MH115347 to K.S.Z.); UK Medical Research Council (N003713 to N.G. and K.L.).

## Author contributions

K.S.Z. and R.G. devised and supervised this study. N.G. supervised aspects of this study. A.H.D.C., A.A.P., L.T., Z.S., S.R., L.H., and K.Y.L. performed experiments, collected, and analyzed data. A.H.D.C., N.G., R.G., and K.S.Z. co-wrote the manuscript.

## Supplementary Material

Supplementary_Figures_bhaa248
