# Supplementary material for: Deficient or Excess Folic Acid Supply During Pregnancy Alter Cortical Neurodevelopment in Mouse Offspring
Source: Cereb Cortex. 2020 Sep 30;31(1):635–49. doi: 10.1093/cercor/bhaa248 (PMC7727343; doi:10.1093/cercor/bhaa248)
Supplement: Supplementary_Figures_bhaa248 [file supplementary_figures_bhaa248.pdf]

Supplementary Figures

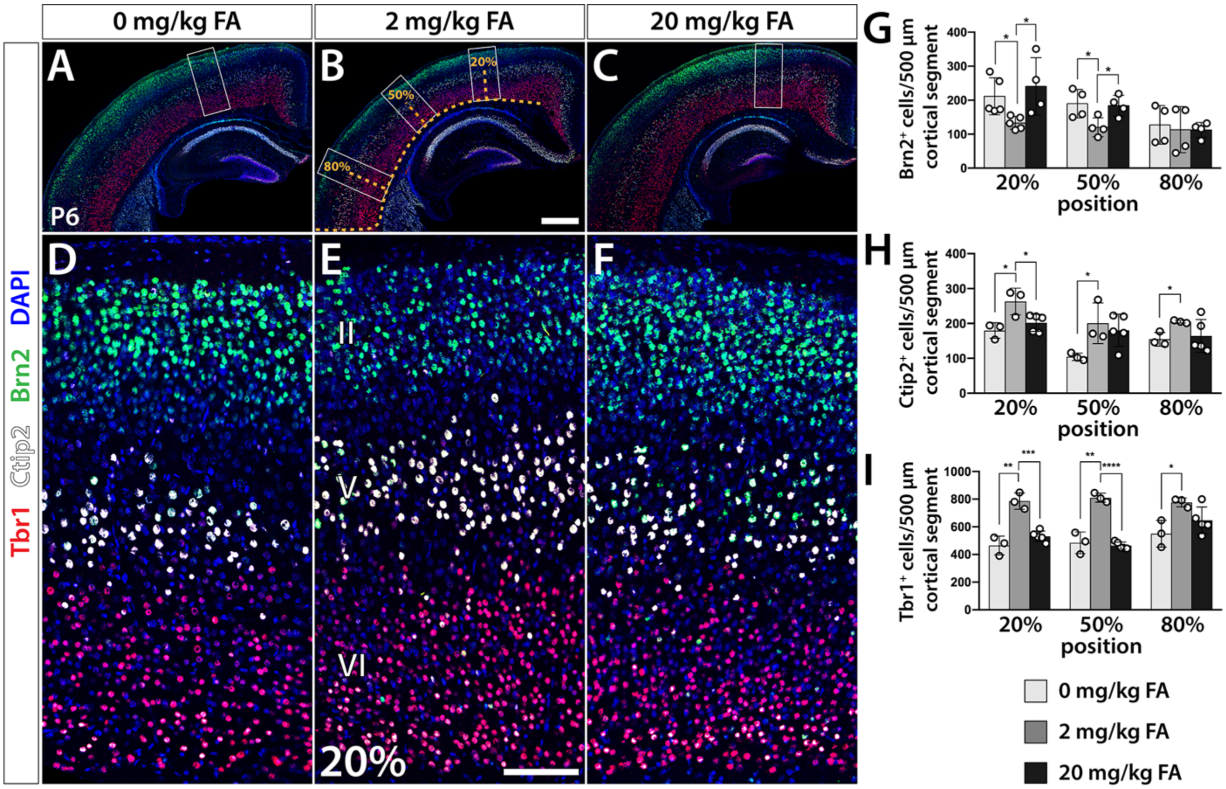

Supplementary Figure 1. Persistent changes in cortical cytoarchitecture in FA deficiency and excess at P6. Immunofluorescent analysis of cortical layer markers Tbr1 (layer VI) and Ctip2 (layer V) reveals significantly reduced numbers for either cell population (*H*, *I*) under conditions of FA deficiency (*A*, *D*, 0 mg/kg chow, light grey in diagram) and FA excess (*C*, *F*, 20 mg/kg chow, black bar) compared with controls (*B*, *E*, 2 mg/kg chow, grey bar). In contrast, Brn2^{+} cells appear increased in numbers. Under either FA condition, dorsomedial (20%) and medial (50%) cortical positions appear more strongly affected than ventrolateral positions (80%). Asterisks indicate significant differences. Cx: cortex, HC: hippocampus. Scale bar in B is 500 µm and in E 100 µm.

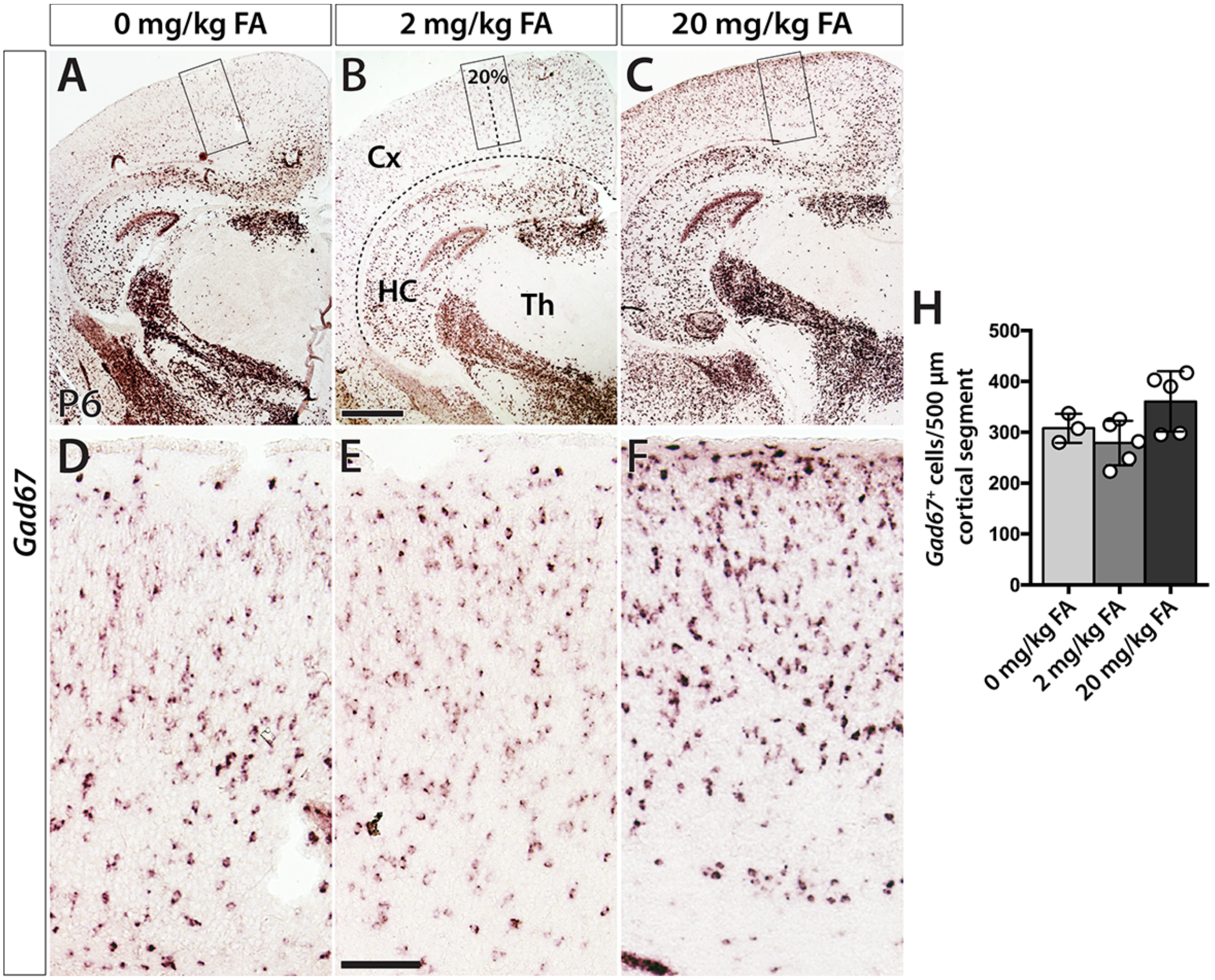

Supplementary Figure 2. No changes in cortical interneuron numbers associated with maternal FA supply. (*A*-*C*) Low-magnification images of *Gad67* RNA *in situ* hybridization analysis is shown on coronal forebrain hemisections of P6 FA deficient (*A*), control (*B*), and FA excess (*C*) offspring. The measured cortical length is indicated by the dashed line in (*B*) and the cortical segments around the 20% position, shown in the high-magnification images below (*D*-*F*), are outlined as rectangular frames. (*H*) Quantitative analysis shows no significant differences between FA test groups and control. Scale bar in B is 500 µm and in E 100 µm. Cx: cerebral cortex, HC: hippocampus, Th: thalamus.

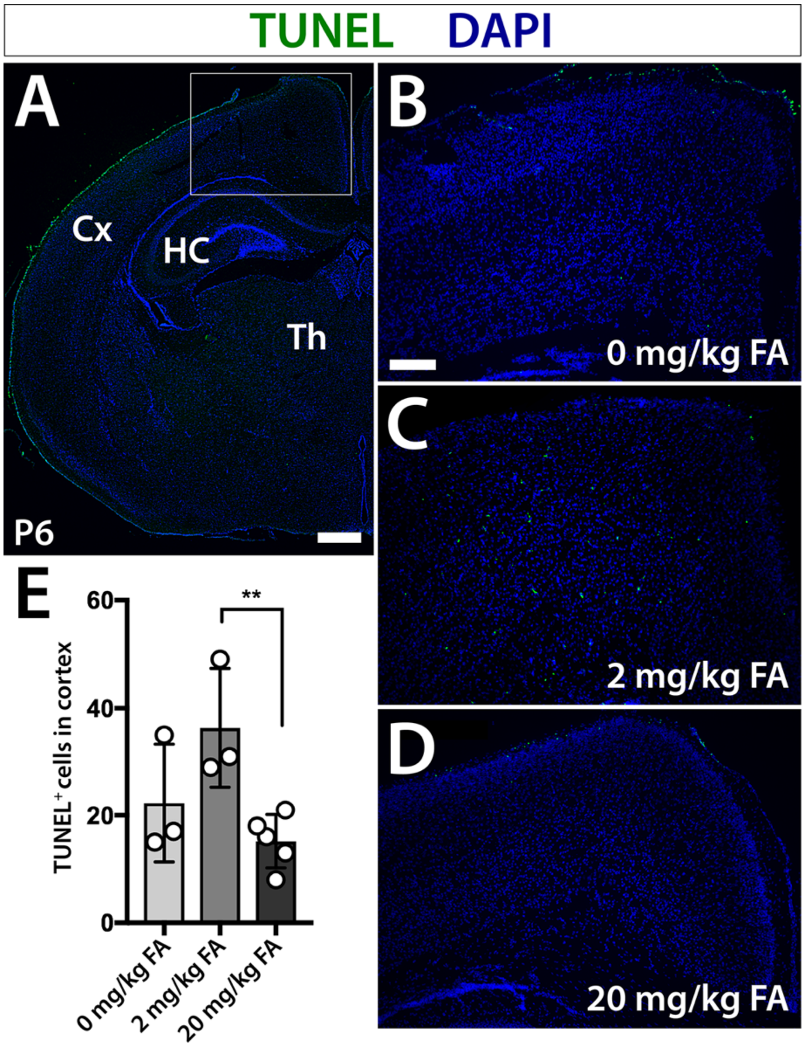

Supplementary Figure 3. Apoptotic rates at P6. TUNEL analysis shows substantially reduced numbers of $TUNEL^+$ cells at P6 compared with earlier stages. (*A*) Low-magnification overview of control brain (2 mg/kg FA). (*B-D*) High-magnification images of cortical dorsomedial aspects of FA deficient (*B*), control (*C*), and FA excess (*D*) offspring. (*E*) Quantification of $TUNEL^+$ cells across the entire neocortex revealed significantly decreased numbers of apoptotic cells in FA excess brains (20 mg/kg FA) compared with controls (2 mg/kg FA). Cx: cortex, HC: hippocampus. Scale bar is 500 μm in A and 200 μm in B.

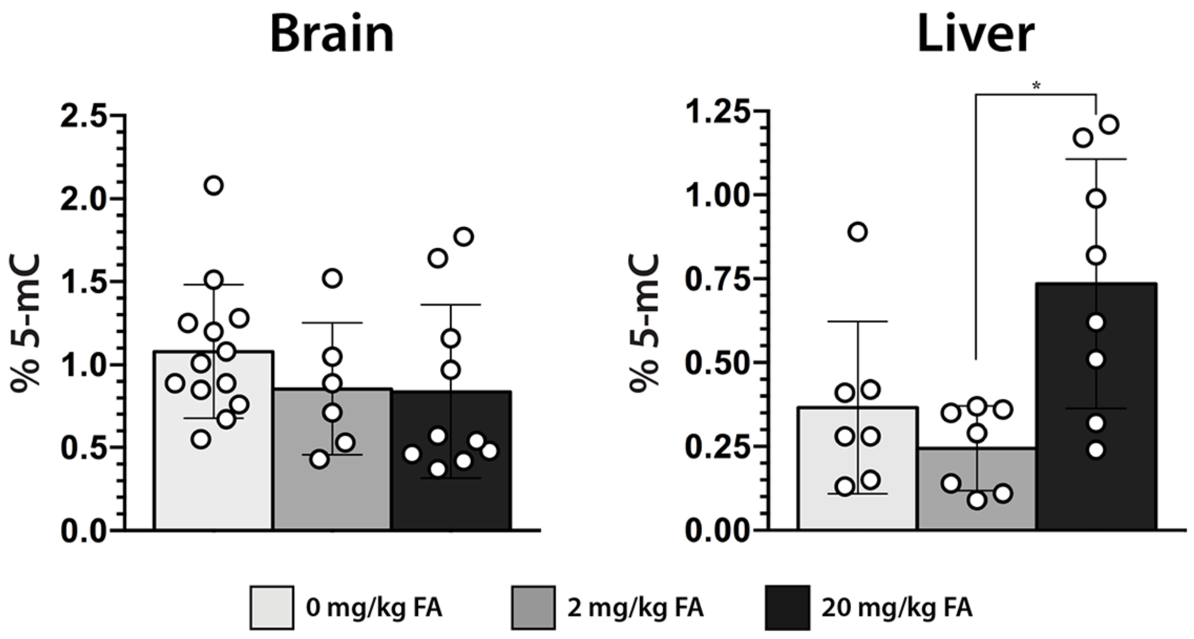

Supplementary Figure 4. Global DNA methylation in brain and liver of FA test and control animals. Bar diagrams depict the mean methylated cytosine percentage (%5-mC) of genomic DNA in P0 brain and liver samples measured by ELISA assay. A significant increase can be observed in liver samples of the FA excess group.

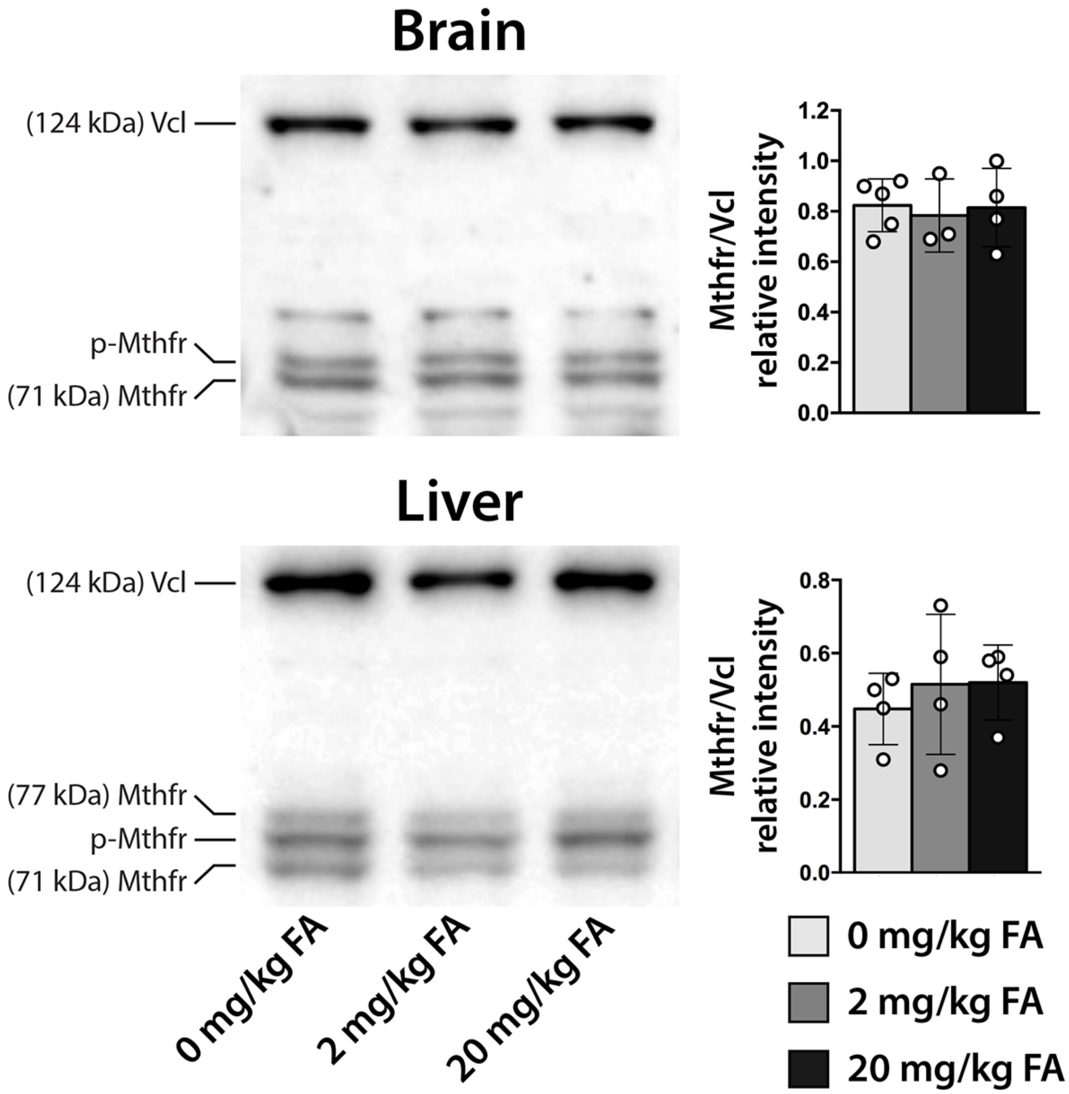

Supplementary Figure 5. Mthfr expression by western blot analysis. Mthfr protein expression by western blot analysis reveals no significant differences in expression levels between FA test groups and control in either P0 brain or liver lysates. In both tissues, Mthfr is detected at 71 kDa and the slightly higher molecular weight phosphorylated p-Mthfr form. In liver, an additional 77 kDa isoform can be detected while brain presented a more complex banding pattern. Vinculin (Vcl) detection was used as internal control and bar diagrams on the right display Mthfr over Vcl relative intensity ratios confirming no significant differences between dietary groups.
